# Short-term blood pressure variability – variation between arm side, body position and successive measurements: a population-based cohort study

**DOI:** 10.1186/s12872-017-0468-7

**Published:** 2017-01-18

**Authors:** Maria Elena Lacruz, Alexander Kluttig, Oliver Kuss, Daniel Tiller, Daniel Medenwald, Sebastian Nuding, Karin Halina Greiser, Stefan Frantz, Johannes Haerting

**Affiliations:** 1 Institute of Medical Epidemiology, Biostatistics and Informatics, Martin-Luther University Halle-Wittenberg, Magdeburger Str. 8, 06112 Halle Saale, Germany; 2 Centre for Health and Society, Faculty of Medicine, Heinrich Heine University Düsseldorf, Düsseldorf, Germany; 3 Institute for Biometrics and Epidemiology, German Diabetes Center, Leibniz Institute for Diabetes Research at Heinrich Heine University Düsseldorf, Düsseldorf, Germany; 4 Department of Medicine III, Martin-Luther University Halle-Wittenberg, Halle Saale, Germany; 5 German Cancer Research Centre, Division of Cancer Epidemiology, Heidelberg, Germany

**Keywords:** (3–5) arm-side, Blood pressure variability, Body position, Hypertension, Successive measurements

## Abstract

**Background:**

Precise blood pressure (BP) measurements are central for the diagnosis of hypertension in clinical and epidemiological studies. The purpose of this study was to quantify the variability in BP associated with arm side, body position, and successive measurements in the setting of a population-based observational study. Additionally, we aimed to evaluate the influence of different measurement conditions on prevalence of hypertension.

**Methods:**

The sample included 967 men and 812 women aged 45 to 83 years at baseline. BP was measured according to a standardized protocol with oscillometric devices including three sitting measurements at left arm, one simultaneous supine measurement at both arms, and four supine measurements at the arm with the higher BP. Hypertension was defined as systolic BP (SBP) ≥140 mmHg and/or diastolic BP (DBP) ≥90 mmHg. Variability in SBP and DBP were analysed with sex-stratified linear covariance pattern models.

**Results:**

We found that overall, no mean BP differences were measured according to arm-side, but substantial higher DBP and for men also higher SBP was observed in sitting than in supine position and there was a clear BP decline by consecutive measurement. Accordingly, the prevalence of hypertension depends strongly on the number and scheme of BP measurements taken to calculate the index values.

**Conclusions:**

Thus, BP measurements should only be compared between studies applying equal measurement conditions and index calculation. Moreover, the first BP measurement should not be used to define hypertension since it overestimates BP. The mean of second and third measurement offers the advantage of better reproducibility over single measurements.

**Electronic supplementary material:**

The online version of this article (doi:10.1186/s12872-017-0468-7) contains supplementary material, which is available to authorized users.

## Background

The indirect blood pressure (BP) measurement by a trained observer is widely used in clinical and epidemiological practice as accurate BP measurement. Office BP has shown a good validity when compared with 24-h ambulatory BP measurements (correlation of 0.9 and 0.8 for systolic BP and diastolic BP respectively) [1]. Precise BP measurements are central for the diagnosis of hypertension and for clinical or epidemiological studies where blood pressure is used as exposure or outcome of interest.

Several factors affect BP measurement, such as environmental factors (i.e. temperature, noise); factors related to the participant (i.e. vigorous physical activity, heavy meal or smoking before measurement); factors related to the device (i.e. cuff size, calibration error) or to the measurement procedure (i.e. left vs. right arm, supine vs. sitting) [2–7]. According to Tolonen et al. [8], these result in variations from 1–2 mmHg up to 20–50 mmHg in individual measurements.

Inter-arm differences in BP vary with the population studied. Some previous studies have suggested a bias towards higher readings from the right arm [9–11], whereas others have failed to show this [12–15]. Additionally, disagreement exists as to whether body position would make any difference in BP readings [6, 16–18]. Several studies have shown that mean supine systolic BP (SBP) is 2–3 mmHg higher and diastolic BP (DBP) 3–5 mmHg lower than sitting BP [6, 16, 17]. However, other studies suggest that posture is unlikely to have significant impact on BP readings [18, 19]. Further, little is known about the magnitude of differences between consecutive BP readings in epidemiologic studies. The results of these studies are conflicting regarding the arm-side, body position and reading order in measured BP (successive measurements in and of itself). Moreover, not only additional unmeasured conditions, but also, already known participants characteristics could further contribute to these inconsistencies. For example, the well-known sex-specific differences in onset and rate of hypertension [20]. Thus an epidemiological study with a highly standardised study protocol, from a sex and age-stratified random sample, measurements conducted by a single trained qualified study nurse and a single weekly checked device could provide helpful information in explaining these uncertainties. Considering the important clinical and epidemiological implications from these differences; i.e., variations in measured BP could lead to variable diagnosis and bias in BP estimates and hypertension frequencies from epidemiological studies, it is important to determine the estimated effect of each of these conditions on the measured BP.

The purpose of this study was therefore, to quantify the variability in BP associated with: a) arm side: right vs left; b) body position: sitting vs supine and c) successive measurements: 1 to 8; in the setting of a population based observational study. Additionally, we aimed to evaluate the influence of different measurement conditions on prevalence of hypertension (defined as SBP above or equal 140 and/or DBP above or equal 90 mmHg).

## Methods

### Participants and setting

The CARLA-Study is a population based cohort study in an elderly population of the city of Halle (Saale) in eastern Germany. Study design and methods were described in detail elsewhere [21]. In brief, subjects were recruited as a random and representative sample from the population registry in a multi-stage process. At baseline 1779 subjects (46% women) aged 45 to 83 years were examined between July 2002 and January 2006 (response rate 64%). The study was in accordance with the declaration of Helsinki. All participants gave their written informed consent. The study was approved by the local ethic commission at the Medical Faculty of the Martin-Luther-University Halle-Wittenberg.

### Outcome

Measurement of BP was conducted within the medical examination after a resting phase of at least five minutes. Systolic and diastolic BP were measured in a sitting position with an oscillometric device (OMRON HEM-705CP, OMRON, Tokyo, Japan) three times on the left arm with a break of three minutes between measurements, one simultaneous supine measurement at both arms, and four supine measurements at the arm with the higher BP (see flow chart, Additional file 1: Figure S1).

Hypertension was within CARLA defined as mean systolic BP (SBP) equal or above 140 mmHg, and/or mean diastolic BP (DBP) above 90 mmHg (defined as the mean of second and third measurement) following [22].

### Statistical analysis

Bland-Altman plots were used to graphically illustrate the variability for the 8 BP measurements. BP values between measurements were evaluated by sex-stratified linear covariance pattern models [23]. The following variables were entered into all of the models as fixed effects (confounders): arm-position (left-sitting, left-supine, right-supine), measurement (1 to 8), and their interaction. A residual ARMA (1,1) (autoregressive moving-average) process was included to account for the additional correlation of BP measurements in the time course within the same participant. All analyses were performed with SAS® 9.3 (SAS Inc., Cary, NC, USA).

## Results

A total of 1779 participants (46% women) took part in the baseline examinations, comparisons could be done among 1743 participants (45% women) with measurements in left and right arm; 1728 (46% women) with measurements in sitting and supine positions and 1778 (46% women) participants with first to third sitting measurements (Table 1). One participant refused to have any BP measurements. Further 36 participants had no supine BP measurements due to contraindication (i.e. thrombose, amputation) or declined participation and for further 15 participants the supine BP measurements had to be stopped before completion.

**Table 1:** Baseline characteristics of the CARLA study population (2002–2006): distribution of cardiovascular risk factors and diseases

	Women			Men		
	N	Median	Min; Q1; Q3; Max	N	Median	Min; Q1; Q3; Max
Age (years)	812	63.3	45.8; 54.9; 71.8; 83.4	967	64.9	45.4; 55.8; 74.0; 83.3
SBP (mmHg)	812	138.0	89.0; 125.5; 155.0; 231.0	966	145.5	93.0; 131.5; 158.5; 227.0
DBP (mmHg)	812	82.5	54.0; 76.0; 90.0; 122.0	966	85.5	52.5; 78.5; 93.0; 127.0
Heart rate (1/min)	778	67.0	39.4; 60.9; 73.4; 105.4	893	65.7	43.9; 60.2; 74.7; 112.4
Total cholesterol (mmol/l)	807	5.7	1.4; 5.0; 6.4; 10.7	960	5.3	2.1; 4.9;5.9; 17.8
HDL cholesterol (mmol/l)	807	1.5	0.4; 1.3; 1.8; 3.4	960	1.2	0.3; 1.0;1.5; 3.0
LDL cholesterol (mmol/l)	801	3.4	0.9; 2.8; 4.0; 7.3	940	3.1	0.9; 2.6;3.7; 7.2
Cholesterol/HDL ratio	807	3.8	1.7; 3.0; 4.5; 9.7	960	4.3	1.7; 3.5; 5.2; 13.0
Triglycerides (mmol/l)	807	1.4	0.2; 1.0; 1.9; 11.6	960	1.7	0.4; 1.2;2.5; 38.2
Glucose (mmol/l)	807	5.4	3.1; 5.1; 5.9; 19.8	960	5.6	3.0; 5.2;6.3; 21.5
HbA_1c_ (%)	807	5.6	3.1; 5.3;5.9; 12.0	960	5.6	2.8; 5.3;6.0; 11.7
Body mass index (kg/m^2^)	812	28.0	18.1; 24.7; 31.5; 53.7	967	27.7	16.5; 25.4;30.8; 43.3
Waist hip ratio	812	0.9	0.7; 0.8; 0.9; 1.2	967	1.0	0.8; 1.0; 1.0; 1.2
For current smokers: pack-years	119	19.5	0.2; 10.6; 26.6; 61.0	225	29.0	0.5; 18.0; 38.4; 85.1
Alcohol consumption gram/day	812	0	0; 0; 5.0; 60.5	964	12.5	0; 2.5; 26.2; 113.6
	N	Proportion	95% CI	N	Proportion	95% CI
Smoking	812			966		
Current	119	14.7	12.2–17.1	225	23.3	20.6–26.0
Past	140	17.2	14.6–19.8	496	51.3	48.2–54.5
Never	553	68.1	64.9–71.3	245	25.4	22.6–28.1
Sports: physically active	347	42.8	39.4–46.2	296	30.6	27.7–33.6
Education years^a^	812			967		
≤10	121	14.9	12.4–17.4	36	3.7	2.5–4.9
11–13	388	47.8	44.3–51.2	391	40.4	37.3–43.5
14–17	231	28.4	25.3–31.6	335	34.6	31.6–37.6
≥18	72	8.9	6.9–10.8	205	21.2	18.6–23.8
Drug use						
Beta-blockers	279	34.4	31.1–37.6	307	31.7	28.8–34.7
Anti-arrhythmics	5	0.6	0.1–1.2	8	0.8	0.3–1.4
ACE-inhibitors	254	31.3	28.1–34.5	340	35.2	32.1–38.2
Diuretics	80	9.9	7.8–11.9	104	10.8	8.8–12.7
Ca-channel blockers	123	15.1	12.7–17.6	148	15.3	13.0–17.6
Disease prevalence						
Myocardial infarction	20	2.4	0.8–3.7	50	5.2	2.7–7.6
Stroke	27	3.3	2.1–4.6	42	4.3	3.1–5.6
Cardiovascular disease^b^	48	5.9	4.3–7.5	153	15.8	13.5–18.1
Hypertension^c^	611	75.2	72.3–78.2	794	82.1	79.7–84.5
Diabetes mellitus^d^	120	14.8	12.3–17.2	154	15.9	13.6–18.3

SBP systolic blood pressure, DBP diastolic blood pressure, HDL high density lipoprotein cholesterol, LDL low density lipoprotein cholesterol, HbA1c glycated hemoglobin, ACE angiotensin converting enzyme, Ca-channel blockers calcium-channel blockers

^a^Education (years of training) according to ISCED classification 1997

^b^CVD: including prevalent myocardial infarction, coronary artery bypass graft (CABG), percutaneous transluminal coronary angioplasty (PTCA), stroke, carotid surgery

^c^Hypertension defined as SBP equal or above 140 and/or DBP equal or above 90 mmHg, and/or use of antihypertensive medication

^d^Diabetes defined as self-reported physician-diagnosed diabetes mellitus and/or use of anti-diabetic medication

**Fig. 1:**
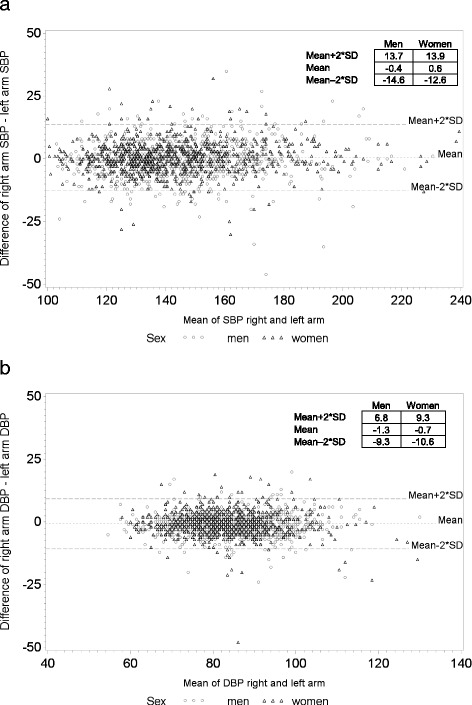
BP arm differences in measurement 4 (supine left and right arm simultaneously). BP differences between right and left arm (right – left) over mean of BP right and left arm for **a** systolic blood pressure (SBP) and **b** diastolic blood pressure (DBP). Black triangles represent women and grey circles men. Mean value (dotted line) and mean values ± 2 standard deviations (SD) (long dash lines) are depicted as horizontal lines

Figure 1 shows that there were no large mean differences in the BP measurements in left vs right arm. Mean differences were for SBP 0.05 mmHg (95% CI (confidence interval) = -0.27 to 0.38) and for DBP −0.98 mmHg (95% CI = -1.19 to−0.77). Though, approximately 10% of the participants had differences greater than 10 mmHg for SBP and about 15% had differences greater than 5 mmHg for DBP. These differences of 10 mmHg for SBP and 5 mmHg for DBP have been previously shown to be clinically relevant [24].

**Fig. 2:**
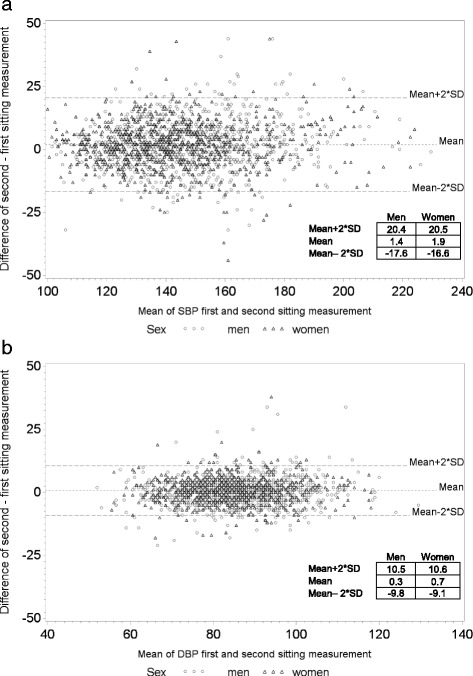
BP successive measurements differences (measurement 1 vs 2). BP differences first and second sitting measurement (first - second) over mean of BP first and second sitting measurement for **a** systolic blood pressure (SBP) and **b** diastolic blood pressure (DBP). Black triangles represent women and grey circles men. Mean value (dotted line) and mean values ± 2 standard deviations (SD) (long dash lines) are depicted as horizontal lines

A further analysis involved the order of the BP measurement (successive measurements): first vs second in sitting (Figure 2). Participants had in average 1.64 mmHg greater SBP (95% CI = 1.20 to 2.08) and 0.51 mmHg greater DBP (95% CI = 0.27 to 0.74) in the first sitting measurement. About 20% of the study participants had differences greater than 10 mmHg for SBP and also 20% had differences greater than 5 mmHg for DBP.

**Fig. 3:**
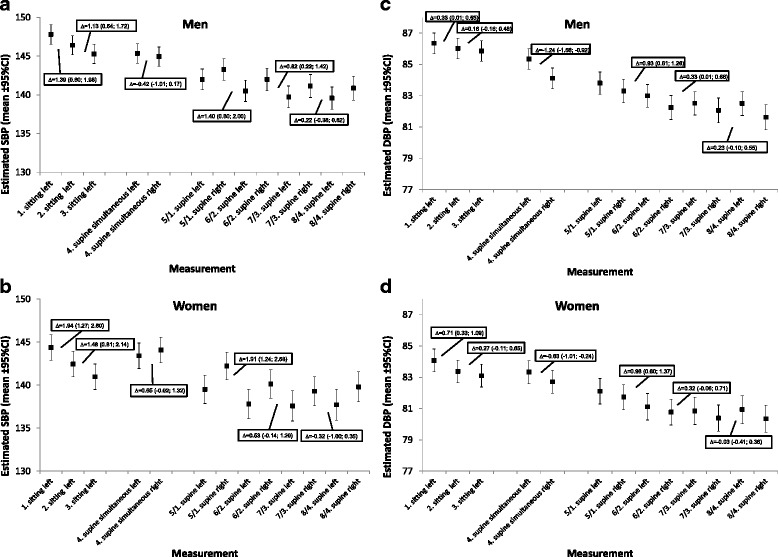
Estimated BP by measurement irrespective of arm-side. Values presented from 5^th^ to 8^th^ measurement are the difference between the mean of one set of right and left measurements and the next set of right and left measurements. Estimated BP with 95%CI and changes in mean estimated BP across measurements for **a** systolic blood pressure (SBP) in men; **b** SBP in women; **c** diastolic blood pressure (DBP) in men; and **d** DBP in women. Δ = difference of the estimated BP measurements; CI = confidence interval

Figure 3 shows the adjusted means with 95% CI for differences in BP in consecutive measurements for men and women respectively. For SBP there are clear differences in sitting position for men and women between first and second and slightly less between second and third measurements, with up to mean differences of 2 mmHg for women between first and second measures. Very similar patterns could be seen for SBP measurements in supine position, with greater differences for women than for men and decreasing in size with further measurements. For DBP, there are also clear differences in sitting position for men and women between first and second and less between second and third measurements, with up to almost 1 mmHg in average for women between first and second measures. However, differences between right and left arm DBP measurements were higher for men, with differences above 1 mmHg. DBP measurements in supine position showed similar patterns for men and women, such that there is a decrease in size with further measurements. The only significant sex difference was seen in the supine measurements were for men there was a decrease in SBP and DBP between second and third measurement, whereas for women no significant decrease could be seen based on the confidence intervals.

Table 2 shows the prevalence of hypertension when considering different BP measurements for men and women. As expected, men had higher prevalence of hypertension irrespective of BP measurement. Consecutive measurements were associated with lower hypertension rates, as SBP and DBP values diminished with further measurements. Supine measurements produced lower hypertension rates. The fact that some participants were classified as hypertensive in each individual measurement, but not with the mean of both measurement (see for example all prevalence estimations for averaged values for men, which are below individual measurement prevalence estimates), can be explained with the fact that in one measurement SBP and in the other DBP determined the hypertensive status, but after averaging both values, none of them were above 140/90 mmHg.

**Table 2:** Prevalence of hypertension by different BP measurements, sex-stratified

	Men			Women		
Sitting (left arm)						
1^st^ measurement	68.7% (*N* = 664)			55.3% (*N* = 535)		
		Mean 1^st^ and 2^nd^	66.5% (*N* = 643)		Mean 1^st^ and 2^nd^	51.7% (*N* = 500)
2^nd^ measurement	66.8% (*N* = 646)			51.2% (*N* = 495)		
		Mean 2^nd^ and 3^rd^	63.2% (*N* = 611)		Mean 2^nd^ and 3^rd^	49.4% (*N* = 478)
3^rd^ measurement	64.0% (*N* = 619)			50.7% (*N* = 490)		
Supine (arm with higher blood pressure)						
1^st^ measurement	57.5% (*N* = 467)			47.9% (*N* = 389)		
		Mean 1^st^ and 2^nd^	53.9% (*N* = 438)		Mean 1^st^ and 2^nd^	45.6% (*N* = 370)
2^nd^ measurement	55.2% (*N* = 448)			44.2% (*N* = 359)		
		Mean 2^nd^ and 3^rd^	51.2% (*N* = 416)		Mean 2^nd^ and 3^rd^	42.2% (*N* = 343)
3^rd^ measurement	51.3% (*N* = 417)			42.9% (*N* = 348)		

## Discussion

The present study sought to quantify the variability in measured BP associated with several factors (arm, position and repetition) and the consequences of body position and successive measurements on the estimation of hypertension prevalence. The main findings are that 1) overall, no mean BP differences were measured according to arm-side; 2) substantial higher DBP and for men also SBP were measured in sitting than in supine position; 3) there was a clear BP decline by consecutive measurement and 4) accordingly, the prevalence of hypertension depends strongly on the body position and successive measurements of BP.

These results replicate previous findings concerning inter-arm differences. Overall, clinically meaningful inter-arm differences were not reproducible. Reductions of 10 mmHg for SBP and 5 mmHg for DBP could be considered clinically relevant [25]. Yet in approximately 10% of the study-participants the inter-arm difference for SBP was above 10 mmHg. This difference can be attributable to random variation but it also could indicate that those subjects suffered from a cardiac disease, e.g. coarcation of the aorta, upper extremity arterial obstruction, dissection or aneurysm of the thoracic aorta [10, 12, 19, 26], and thus the inter-arm difference could mask treatment effects. Unfortunately, this hypothesis cannot be tested, as this accurate diagnostic information is not available for the study population. Nevertheless, in a population-based cohort and a patient-based cohort, it has been shown that there was no association between short-term blood pressure variability and subclinical target organ damage [27, 28].

The substantially higher DBP in sitting than in supine position is in agreement with some previous studies [6, 16]. The finding of higher SBP in sitting than in supine position however, dissents with those studies [6, 16]. This disagreement could be due to different protocols used in the studies mentioned above, i.e. same arm-position for measurements in sitting and supine position could lead to lower differences among readings. A further influencing factor could be the measurement order, which in our study was fixed starting with participants in sitting position. Thus the position effect cannot be separated from the successive measurements effect.

The number of readings was shown to have systematically affected the BP measurement. Compared to previous studies, we found a smaller decline from first to second sitting measurement but a more consistent decline to the third measurement [29]*.* There were no further reductions after the third measurement in supine (almost no difference between third and fourth measurements); thus, it seems that at that point stability was achieved. There was a similar fall in the estimated prevalence of hypertension. This decline occurred regardless of adjustment for confounders which showed to be related to the BP decline: baseline BP level, age and BMI [29]. Analysis with further adjustment for those confounders led to almost identical results (data not shown). The first measurement clearly surpassed following BP measurements. This fact could be due to a combination of white coat hypertension, blood pressure variability and/or device accuracy [30]. Thus, the use of a single measurement introduces a systematic error that could be corrected by excluding the first reading. Accordingly, BP estimates in studies obtained from several readings are usually lower than those obtained from a single reading [6, 29, 31]. In the clinical praxis, the uncertainty about the true BP values has as a consequence fluctuating treatment decisions [32]. Therefore, guidelines for clinical practice recommend diagnosing hypertension based on multiple measurements at different visits. For epidemiological research, there are several difficulties (i.e. logistic and financial) associated with two-visit BP measurements, which lead to a single visit strategy. On the other hand, single visit measurements may lead to erroneous conclusions in the interpretation of prevalence rates, awareness, and medical control of hypertension in different countries [33–35]. Interestingly, it has been shown that misclassification was more common in younger (<30 years) than older participants [36], with rates varying from 12% in subjects aged 62 ± 11 years [37], to 35% in subjects aged 39 ± 9 years and 15 to 69 years [36, 38], respectively.

There is evidence that an increased visit-to-visit variability in SBP is a powerful predictor of end-organ damage and cognitive decline [39–41]. Moreover, patients with controlled mean BP but high visit-to-visit SBP variability had an increased risk of stroke than those with low SBP variability [42]. Previous studies have shown visit-to-visit SBP variability to be strongly influenced by drug-class effects with increased variability in participants medicated with β-blockers [43]. We tried to replicate these findings with short-term SBP variability, in stratified models as well as in models with further adjustment for medication as covariate (Additional file 2: Table S1) and found no effect of medication use or medication class (β-blockers or Angiotensin converting enzyme (ACE) inhibitors) related to SBP or DBP variability. BP variability results from extrinsic and intrinsic regulatory mechanisms and substantially differs in clinical significance and prognostic implications depending on the time interval considered for its assessment [44].

It has been speculated that the lack of standardization in BP measurement procedure can lead to clinically relevant differences in estimations of prevalence and incidence of hypertension [45]. Thus, it is possible that it also influences the estimates of association studies between hypertension and other factors. Therefore, published prevalence of hypertension should clearly be accompanied by a definition of the measurement procedure: arm-side, body position, number of readings and its combination for the definition of hypertension.

The present study has several important strengths. Foremost, it is a population-based sample, with well-defined health outcomes. The results of this study may be considered representative for the general population aged 45 to 85 years since a random sample from the population registry of the city of Halle had been selected, and a high participation rate could be achieved. Some limitations, however, need to be addressed. Due to practicability reasons, as this study was framed in the examination part of an epidemiological cohort, there were some restrictions to BP measurements: 1) there was only one simultaneous supine measurement; 2) supine measurements were not consistently throughout on one arm, but in that with higher BP. This was done following the recommendation for clinical BP measurement, that in case of disparity, the arm with higher pressure should be used for subsequent readings.

Moreover, there might be problems to separate posture effect from measurement effect. We approached this problem by including all measurements in a common statistical model with proper adjustment for measurements within the same subject. However, not all combinations of factors are observed (actually constituting an incomplete block design) and we have to rely on model extrapolations for some of the reported differences. Thus slight differences measured in sitting vs supine positions (mean difference for SBP was 4.05 mmHg and for DBP was 2.98 mmHg greater in sitting) could be due to successive measurements instead of body position.

A further question is how much of the BP variability can be due to the measuring device. It has been reported that the OMRON HEM-705CP device fulfils the recommendation criteria of the international protocol [46]. Finally, different resting times were used for sitting and supine readings (3 and 1 min respectively).

## Conclusions

In conclusion, the measured BP strongly depends on position, successive measurements and combination of subsequent BP readings. Thus, BP measurements should only be compared between studies applying equal measurement conditions. Moreover, the first BP measurement should not be used to define hypertension since it overestimates mean individual BP. The mean of second and third measurement offers the advantage of better reproducibility over single measurements.

## Additional files

Additional file 1: Figure S1. Flow chart of the study design. (PPT 176 kb)
Additional file 2: Table S1. Estimated BP by measurement stratified by medication use. (DOCX 19 kb)

## Abbreviations

ACE: Angiotensin converting enzyme
ARMA: Autoregressive moving-average
BP: Blood pressure
Ca-channel blockers: Calcium-channel blockers
CI: Confidence interval
CVD: Cardiovascular disease
DBP: Diastolic blood pressure
HbA1c: Glycated hemoglobin
HDL: High density lipoprotein cholesterol
LDL: Low density lipoprotein cholesterol
SBP: Systolic blood pressure
