# Supplementary material for: Short-term blood pressure variability – variation between arm side, body position and successive measurements: a population-based cohort study
Source: BMC Cardiovasc Disord. 2017 Jan 18;17:31. doi: 10.1186/s12872-017-0468-7 (PMC5241970; doi:10.1186/s12872-017-0468-7)
Supplement: Additional file 1: Figure S1. — Flow chart of the study design. (PPT 176 kb) [file 12872_2017_468_MOESM1_ESM.ppt]

## Slide 1
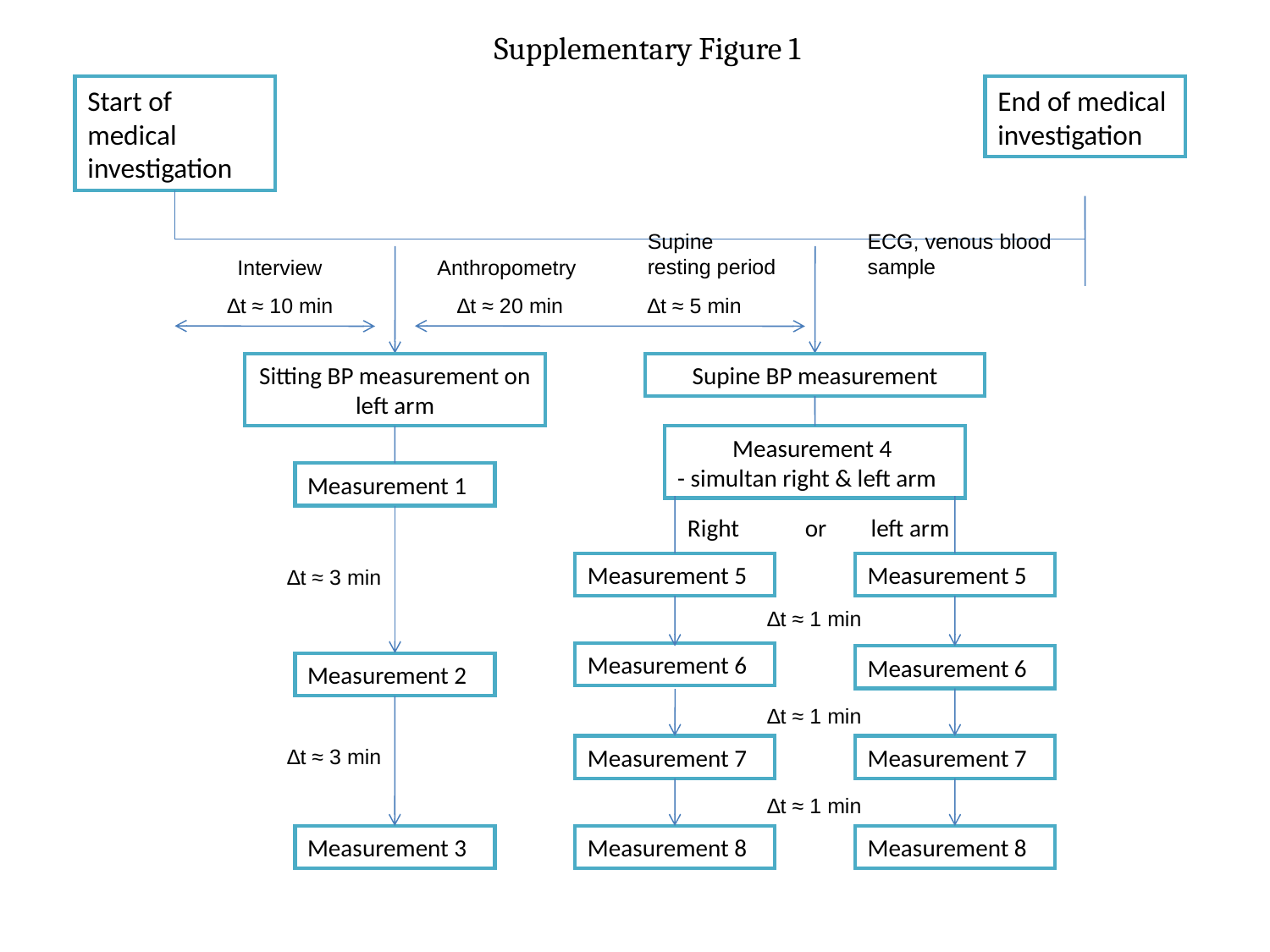

Supplementary Figure 1
Start of medical investigation
End of medical investigation
Supine resting period
ECG, venous blood sample
Interview
Anthropometry
∆t ≈ 10 min
∆t ≈ 20 min
∆t ≈ 5 min
Sitting BP measurement on left arm
Supine BP measurement
Measurement 4
- simultan right & left arm
Measurement 1
Right or left arm
Measurement 5
Measurement 5
∆t ≈ 3 min
∆t ≈ 1 min
Measurement 6
Measurement 6
Measurement 2
∆t ≈ 1 min
Measurement 7
Measurement 7
∆t ≈ 3 min
∆t ≈ 1 min
Measurement 3
Measurement 8
Measurement 8
