# Supplementary material for: Short-term blood pressure variability – variation between arm side, body position and successive measurements: a population-based cohort study
Source: BMC Cardiovasc Disord. 2017 Jan 18;17:31. doi: 10.1186/s12872-017-0468-7 (PMC5241970; doi:10.1186/s12872-017-0468-7)
Supplement: Additional file 2: Table S1. — Estimated BP by measurement stratified by medication use. (DOCX 19 kb) [file 12872_2017_468_MOESM2_ESM.docx]

**Supplementary table 1. Estimated BP by measurement stratified by medication use.**

|  |  | **Beta-blockers (n=586)*** | | | **ACE (n=594)*** | | | **no medication (n=794)** | | |
| --- | --- | --- | --- | --- | --- | --- | --- | --- | --- | --- |
|  | **Measurement** | **Mean** | **95%CI** | | **Mean** | **95%CI** | | **Mean** | **95%CI** | |
| **SBP** | **1. sitting** | 147.2 | 148.3 | 146.1 | 149.6 | 150.7 | 148.5 | 142.9 | 143.6 | 142.2 |
|  | **2. sitting** | 145.7 | 146.8 | 144.6 | 147.3 | 148.5 | 146.2 | 141.5 | 142.2 | 140.8 |
|  | **3. sitting** | 143.5 | 144.6 | 142.4 | 145.9 | 147.1 | 144.8 | 140.1 | 140.8 | 139.4 |
|  | **4. supine left** | 147.0 | 148.1 | 145.8 | 148.5 | 149.7 | 147.4 | 140.5 | 141.2 | 139.8 |
|  | **4. supine right** | 147.2 | 148.4 | 146.1 | 148.7 | 149.8 | 147.6 | 140.5 | 141.2 | 139.8 |
|  | **5/1. supine left** | 142.7 | 144.0 | 141.3 | 144.3 | 145.7 | 143.0 | 137.7 | 138.6 | 136.9 |
|  | **5/1. supine right** | 144.2 | 145.5 | 142.8 | 146.8 | 148.1 | 145.4 | 139.2 | 140.1 | 138.4 |
|  | **6/2. supine left** | 140.6 | 142.0 | 139.2 | 141.8 | 143.2 | 140.4 | 136.6 | 137.5 | 135.8 |
|  | **6/2. supine right** | 142.4 | 143.8 | 141.0 | 144.4 | 145.9 | 142.9 | 137.6 | 138.5 | 136.7 |
|  | **7/3. supine left** | 139.7 | 141.2 | 138.2 | 140.8 | 142.2 | 139.3 | 136.7 | 137.6 | 135.8 |
|  | **7/3. supine right** | 141.1 | 142.6 | 139.6 | 143.2 | 144.7 | 141.7 | 137.2 | 138.1 | 136.3 |
|  | **8/4. supine left** | 139.4 | 140.9 | 137.9 | 141.3 | 142.8 | 139.8 | 136.8 | 137.8 | 135.9 |
|  | **8/4. supine right** | 140.9 | 142.4 | 139.4 | 142.6 | 144.1 | 141.0 | 137.8 | 138.7 | 136.8 |
| **DBP** | **1. sitting** | 83.8 | 84.3 | 83.2 | 83.8 | 84.3 | 83.2 | 86.5 | 86.9 | 86.2 |
|  | **2. sitting** | 83.5 | 84.1 | 83.0 | 83.5 | 84.1 | 83.0 | 85.9 | 86.3 | 85.5 |
|  | **3. sitting** | 83.0 | 83.6 | 82.5 | 83.0 | 83.6 | 82.5 | 85.9 | 86.3 | 85.6 |
|  | **4. supine left** | 84.1 | 84.7 | 83.6 | 84.1 | 84.7 | 83.6 | 84.7 | 85.1 | 84.3 |
|  | **4. supine right** | 83.0 | 83.5 | 82.4 | 83.0 | 83.5 | 82.4 | 83.9 | 84.2 | 83.5 |
|  | **5/1. supine left** | 82.8 | 83.4 | 82.1 | 82.8 | 83.4 | 82.1 | 83.5 | 84.0 | 83.1 |
|  | **5/1. supine right** | 81.8 | 82.4 | 81.1 | 81.8 | 82.4 | 81.1 | 83.3 | 83.8 | 82.9 |
|  | **6/2. supine left** | 81.6 | 82.3 | 80.9 | 81.6 | 82.3 | 80.9 | 83.2 | 83.6 | 82.7 |
|  | **6/2. supine right** | 80.5 | 81.3 | 79.8 | 80.5 | 81.3 | 79.8 | 82.4 | 82.9 | 82.0 |
|  | **7/3. supine left** | 81.2 | 81.9 | 80.4 | 81.2 | 81.9 | 80.4 | 82.7 | 83.2 | 82.3 |
|  | **7/3. supine right** | 79.9 | 80.6 | 79.1 | 79.9 | 80.6 | 79.1 | 82.2 | 82.7 | 81.8 |
|  | **8/4. supine left** | 81.2 | 81.9 | 80.4 | 81.2 | 81.9 | 80.4 | 82.9 | 83.4 | 82.4 |
|  | **8/4. supine right** | 79.4 | 80.2 | 78.7 | 79.4 | 80.2 | 78.7 | 82.1 | 82.6 | 81.6 |

*numbers do not add up to 1779, because 195 participants took beta-blockers and ACE-inhibitors.
